# Antimicrobial Stewardship in Acute-Care Hospitals: A Report of the California Healthcare-Associated Infections Honor Roll

**DOI:** 10.1017/ash.2021.59

**Published:** 2021-07-29

**Authors:** Jane Kriengkauykiat, Erin Epson, Erin Garcia, Kiya Komaiko

## Abstract

**Background:** Antimicrobial stewardship has been demonstrated to improve patient outcomes and reduce unwanted consequences, such as antimicrobial resistance and *Clostridioides difficile* infection. The California Department of Public Health (CDPH) Healthcare-Associated Infection (HAI) Program developed an honor roll to recognize facilities with the goal of promoting antimicrobial stewardship programs and encouraging collaboration and research. **Methods:** The first open enrollment period in California was from August 1 to September 1, 2020, and was only open to acute-care hospitals (ACHs). Enrollment occurs every 6 months. Applicants completed an application and provided supporting documentation for bronze, silver, or gold designations. The criteria for the bronze designation were at least 1 item from each of CDC’s 7 core elements for ACHs. The criteria for silver were bronze criteria plus 9 HAI program prioritized items (based on published literature) from the CDC Core Elements and demonstration of outcomes from an intervention. The criteria for gold designation were silver criteria plus community engagement (ie, local work or collaboration with healthcare partners). Applications were evaluated in 3 phases: (1) CDPH reviewed core elements and documentation, (2) CDPH and external blinded antimicrobial stewardship experts reviewed outcomes as scientific abstracts, and (3) CDPH reviewed each program for overall effectiveness in antimicrobial stewardship and final designation determination. Designations expire after 2 years. **Results:** In total, 119 applications were submitted (30% of all ACHs in California), of which 100 were complete and thus were included for review. Moverover, 33 facilities were from northern California and 67 were from southern California. Also, 85 facilities were part of a health system or network, 14 were freestanding, and 1 was a district facility. Facility types included 68 community hospitals, 17 long-term acute-care (LTAC) facilities, 17 academic or teaching hospitals, 4 critical-access hospitals, and 4 pediatric hospitals. There was an even distribution of hospital bed size: 35 facilities had <250 beds. The final designations included 19 gold, 35 silver and 43 bronze designations. There was 44% incongruency in applicants not receiving the designation for which they applied. Community hospitals were 63%–74% of all designations, and no LTACs received a gold designation. Moreover, 63% of hospitals with gold designations had >250 beds, and 47% of hospitals with bronze designations had <1 25 beds. **Conclusions:** The number of applicants was higher than expected because the open enrollment period occurred during the COVID-19 pandemic. This finding demonstrates the high importance placed on antimicrobial stewardship among ACHs. It also provides insight into how facilities are performing and collaborating and how CDPH can support facilities to improve their ASP.

**Funding:** No

**Disclosures:** None

